# A systematic review on the role of biosecurity to prevent or control colibacillosis in broiler production

**DOI:** 10.1016/j.psj.2024.103955

**Published:** 2024-06-06

**Authors:** G. Tilli, R. Vougat Ngom, H. Cardoso de Carvalho Ferreira, I. Apostolakos, S. Paudel, A. Piccirillo

**Affiliations:** ⁎ Department of Comparative Biomedicine and Food Science, University of Padua, Legnaro, Italy; † Department of Animal Production, School of Veterinary Medicine and Sciences, University of Ngaoundéré, Ngaoundéré, Cameroon; ‡ Veterinary Public Health Institute, Vetsuisse Faculty, University of Bern, Bern, Switzerland; § Flanders Research Institute for Agriculture, Fisheries and Food, Merelbeke, Belgium; # Dairy Research Institute, Hellenic Agricultural Organization “DIMITRA”, Ioannina, Greece; || Clinic for Poultry and Fish Medicine, Department for Farm Animals and Veterinary, Public Health, University of Veterinary Medicine Vienna, Vienna, Austria; ¶ Department of Infectious Diseases and Public Health, Jockey Club College of Veterinary, Medicine and Life Sciences, City University of Hong Kong, Kowloon, Hong Kong

**Keywords:** biosecurity, broiler, colibacillosis, control, prevention

## Abstract

This systematic review aimed at investigating the role that biosecurity can have in preventing or controlling colibacillosis in broiler production. Primary studies with natural or experimental exposure to avian pathogenic *Escherichia coli*, evaluating any biosecurity measure to prevent or control colibacillosis in broiler chickens with at least one of the following outcomes: feed conversion ratio (**FCR**), condemnations at slaughter, and mortality due to colibacillosis, were included. A systematic search was carried out in 4 databases according to the Cochrane handbook and reported following the PRISMA 2020 directions. Studies (n = 3,886) were screened in a 2-phase process and data matching the inclusion criteria were extracted. Risk of bias assessment was performed. Four studies reporting biosecurity measures to prevent or control colibacillosis in broiler production were included. In all studies, only disinfection during either the pre-hatching period (n = 3) or the post-hatching period (n = 1) was evaluated as biosecurity measure in broiler production, as well as its effect on FCR (n = 2) and mortality (n = 4) due to colibacillosis. No studies with effects on condemnations at slaughter were found. Due to the heterogeneity of studies in regard to interventions and outcomes, meta-analysis was not carried out. The limited findings of this systematic review do not provide a comprehensive evidence to statistically evaluate the efficacy of biosecurity to prevent or control colibacillosis in broiler production. The scarcity of evidence found suggests that further and deeper investigations on the topic are needed, considering the variety of interventions related to biosecurity.

## INTRODUCTION

Colibacillosis in poultry production refers to a set of infections/diseases caused by virulent strains of *Escherichia coli*, namely avian pathogenic *Escherichia coli* (**APEC**) (Guabiraba, Schouler, 2015; Kathayat et al., 2021). Contrary to the commensal *E. coli*, APEC can act as a primary or secondary pathogen, affecting the host mainly when the immune system is compromised (Nolan et al., 2020). Colibacillosis can manifest as either a localized or a systemic infection, resulting in various diseases that can affect all poultry species (Kathayat et al., 2021). APEC infections can lead to severe clinical manifestations including colisepticemia, airsacculitis, cellulitis, omphalitis, peritonitis and salpingitis representing a common issue in worldwide poultry production (Newman et al., 2021).

Infection of poultry with APEC can occur at different stages of the production chain (i.e., hatcheries, transport, rearing cycle) (Poulsen et al., 2017), with meat-production chickens being more susceptible during the rearing phase compared to other poultry species (Landman and Van Eck, 2015), and with a high prevalence of APEC-related infections in the meat-producing industry (Apostolakos et al., 2021). Thus, economic losses due to APEC infections in the poultry industry are estimated in millions of dollars worldwide (Guabiraba and Schouler, 2015; Ozaki et al., 2017; Mehat et al., 2021). Specifically, Landman and van Eck (2015) estimated the economic impact of peritonitis syndrome due to APEC to be 3.3 million euros in the Dutch poultry meat-producing sector, mainly related to mortality (culling of dead birds) and antibiotic costs.

To date, several strategies have been developed to prevent or control colibacillosis. They include the use of antibiotics, vaccination, management practices, biosecurity measures, nutritional modulations and nutraceuticals, as well as probiotics, bacteriophages, and other alternatives against APEC infections (i.e., innate immune stimulants, APEC virulence and growth inhibitors, antimicrobial peptides) (Helmy et al., 2018; Nguyen et al., 2021; Sarfraz et al., 2022; Paudel et al., 2024). Prevention strategies should avoid an increased prophylactic use of antibiotics (Eijck and De Wilt, 2009), as this can lead to the development of antimicrobial resistance. On the other hand, biosecurity is a key prevention strategy to reduce the incidence of disease. Therefore, implementation of biosecurity should be encouraged as preventive measure to protect the flock against the introduction and spread of pathogens (WOAH, 2022).

Since the introduction of *E. coli* may occur at different steps of the production chain, there are several critical points in which biosecurity measures may have a relevant prevention role for colibacillosis onset in the flock. Specifically, the most critical ones are: the correct management of the broiler breeder flocks to avoid vertical transmission (Giovanardi et al., 2005), the correct practices in the hatcheries as they may represent a source of APEC (Ozaki et al., 2017; Zhao et al., 2018), as well as the typology (i.e., nest eggs vs. floor eggs) of eggs used for hatching. Ahamed et al. (2019) and Christensen et al. (2021) showed that nest eggs are associated with a lower *E. coli* colonization of chicks and have a positive effect on hatchability rate in the hatcher when compared to floor eggs. A proper attention on biosecurity measures should be also paid during the rearing cycle to avoid horizontal transmission. For example, potential *E. coli* transmission routes like insects (Schiavone et al., 2020; Tamburro et al., 2022) or rodents (Himsworth et al., 2016) should be considered.

The available literature depicts an empirical-based common understanding of the importance of biosecurity measures in colibacillosis control in poultry production (Lutful Kabir, 2010; Christensen et al., 2021). However, to the best of the authors’ knowledge there is no evidence on the efficacy of biosecurity implementation in the prevention or control of poultry colibacillosis. Therefore, the link between biosecurity and colibacillosis still needs to be fully elucidated, especially considering the worldwide concern for this disease in poultry production.

The aim of this study was, therefore, to provide scientific evidence on the efficacy of biosecurity in broilers at risk of colibacillosis through a systematic review process performed within the COST Action CA18217 - European Network for Optimization of Veterinary Antimicrobial Treatment (**ENOVAT**). Specifically, the objective of this systematic review was to address the following research question: “In broilers at risk of colibacillosis, does biosecurity versus no biosecurity result in higher feed conversion ratio (**FCR**)/fewer condemnations at slaughter/lower mortality?”.

## MATERIALS AND METHODS

This systematic review was performed as described in the Cochrane handbook for systematic reviews of interventions method (Higgins et al., 2022), and reported according to the Preferred Reporting Items for Systematic reviews and Meta-Analyses (**PRISMA**) 2020 statement (Page et al., 2021).

### Protocol and Registration

An *a priori* protocol was developed, stored at Padua Research Archive institutional repository (available at: https://www.research.unipd.it/handle/11577/3439978) and then registered in the Systematic Reviews for Animals and Food (**SYREAF**) website (available at: https://syreaf.org/protocols/).

### Eligibility Criteria

Primary research studies eligible for inclusion were screened according to the following PICO: studies conducted in broiler chickens (Population) evaluating a biosecurity measure (Intervention) compared to lower levels or absence of biosecurity measures (Comparator) to prevent or control colibacillosis. The included studies measured and reported the results of at least one of the following outcomes: feed conversion ratio (**FCR**), condemnations at slaughter, and mortality due to colibacillosis (Outcome). The choice of the PICO and the definition of the search strings were based on *a priori* expert consultation. In addition, only randomized control trials with natural or experimental exposure to APEC, published in English and/or Spanish were eligible for inclusion. Limitations on the publication date or geographical location of the studies were not applied.

### Information Sources

Bibliographic databases that provided a high level of article recall across biomedical articles were used (Bramer et al., 2017). Specifically, the searched databases were: CAB Abstracts (in Ovid) and Agricola (in ProQuest) accessed via the University of Bern (Switzerland); MEDLINE (in PubMed) and Web of Sciences (**WOS**) accessed via the University of Padova (Italy). Within WOS, Web of science core collection database was used, except for Social Sciences Citation Index (**SSCI**), Arts & Humanities Citation Index (**AHCI**), Conference Proceedings Citation Index-Social Science & Humanities (**CPCI-SSH**), Book Citation Index - Science (**BKCI-S**) and Book Citation Index - Social Sciences & Humanities (**BKCI-SSH**) as their research focus was not within the scope of this review. In addition to these databases, reference lists of the included studies (after full text screening) and review papers were screened. Google Scholar was used for backward search starting from the included papers.

### Search Strategy

The search strategy involved a multi-stranded approach that uses a series of searches, with different combinations of concepts to gather all possibilities and therefore achieve high sensitivity (Higgins et al., 2022). The search string formatting was modified as needed to reflect differences in database interfaces for each of the selected ones. Search strategy included the following concept related to the PICO: [Broilers] AND [Biosecurity] AND [Colibacillosis]. Table 1 shows the adopted search strategy for WOS database, those adopted for the remaining databases are provided as Supplementary Table 1 and 2 for MEDLINE, 3 for Agricola and 4 for CAB Abstracts.

**Table 1:** Full search string used to retrieve studies examining the efficacy of biosecurity to prevent or control colibacillosis in broiler production as applied in Web of Science (via Web of Science).

	Search 1 - November 1st 2021	Search 2 - April 13th 2023
TS = (“chicken*” OR “poultry*” OR “flock*” OR “gallus” OR “broiler*”) AND	195,332	16,927
TS = (“Biosecurity” OR “Clean*” OR “Disinfect*” OR “Disinfest*” OR “Pest ” OR “Insect*” OR “Vermin*” OR “Rodent*” OR “Fomites ” OR “Sanit*” OR “Hygien*” OR “All in-all out” OR “Downtime” OR “Turnaround” OR “Biological break” OR “Filter zone ” OR “Danish entry system” OR “Footdips” OR “Visitor*” OR “Thinning” OR “Depopulation”) AND	949,452	101,694
TS = (“colibacillosis” OR “colisepticaemia” OR “peritonitis” OR “coli” OR “Escherichia” OR “coliform” OR “colisepticemia” OR “coligranuloma” OR “Hjarre's” OR “air sac disease” OR “cellulitis” OR “osteomyelitis” OR “brittle bone disease” OR “salpingitis” OR “synovitis” OR “omphalitis” OR “enteritis” OR “hemorrhagic septicemia” OR “chronic respiratory disease” OR “swollen head syndrome” OR “venereal colibacillosis” OR “coliform cellulitis” OR “yolk sac infection” OR “APEC” OR “pathogenic E. coli” OR “primary infection” OR “secondary infection” OR “multifactorial” OR “multicausal”)	650,634	49,789
#1 AND #2 AND #3	1,264	299

Search strategies adopted for the remaining databases are provided as SM 1 and 2 for MEDLINE, 3 for Agricola and 4 for CAB Abstracts. Dates of the original search: November 1st 2021; date of the additional search: April 13th 2023.

Searches were conducted twice applying the same search strategy: the original search was conducted in November 2021 (papers published to that date), followed by a second search conducted in April 2023 (studies published from November 2021 to that date). The search was conducted twice as an attempt to retrieve potential new records published in the new timeframe. For both searches, information sources were the same.

### Selection Process

Search results were uploaded in Zotero software (version 6.0.26), while duplicates and retracted citations were removed. After this, citations were uploaded in Rayyan software (https://www.rayyan.ai/) for the two-step screening. The whole screening process (both title and abstract, and full text) was carried out by 2 independent reviewers. Citations were excluded if both reviewers responded “NO” to any of the screening questions. When consensus between the two reviewers was not reached, a third reviewer was asked to solve the conflict.

The first step consisted of title and abstract screening. At the beginning, the concordance among all the 3 reviewers was evaluated by screening 100 randomly selected studies. This calibration exercise enabled discussion and solved disagreements before carrying out the full selection process (Sanguinetti et al., 2021). At this stage, eligibility of studies was assessed with the following questions:

1. Is the study primary research assessing the use of biosecurity measure(s) to prevent or control colibacillosis in broiler production chain? Yes [Pass], No [Exclude], Unclear [Pass]
2. Does the study include an eligible comparator *via* a controlled trial, disease challenge study or observational study? Yes [Pass], No [Exclude], Unclear [Pass]

Studies that met these inclusion criteria passed to the second step which consisted of full text screening. As in the previous step, the calibration exercise was carried out on 25 randomly selected papers. Eligibility of studies was assessed with the following questions:

1. Is a full text of more than 500 words available? Yes [Pass], No [Exclude]
2. Is a full text available in English or Spanish? Yes [Pass], No [Exclude]
3. Is the Population of the study broilers? Yes [Pass], No [Exclude], Unclear [Exclude]
4. Is the Intervention of the study the use of biosecurity measure(s) to prevent or control colibacillosis in broilers? Yes [Pass], No [Exclude], Unclear [Exclude]
5. Is at least one of mortality, FCR, or condemnations due to colibacillosis the Outcome(s) described? Yes [Pass], No [Exclude]
6. Is the study design a controlled trial with natural or experimental disease exposure? Yes [Pass], No [Exclude]

### Data Collection Process

A Microsoft Excel standardized spreadsheet was used for data extraction. This datasheet was created by one of the authors and validated by all the others. Two independent reviewers performed the data extraction from the included studies. Since few studies were included, no calibration exercise was performed. After data collection from all the papers that met the full text screening criteria, the 2 reviewers validated the extracted data. Validation occurred through discussion among the authors.

### Data Items

*Study Characteristics.* Data extracted included general information on the study and information on the population and the intervention, specifically: year and country (where the trial was conducted); duration of the study (days); and study design (cross sectional or longitudinal study). Data related to the population included: production stage (hatchery, farm, not defined); population (eggs, chicks); age of the population (days) when intervention was applied; flock typology (commercial or experimental flocks); birds’ size, origin, breed, and sex; production type (conventional, organic, antibiotic-free); birds’ production stage/age when outcome(s) were measured (day, d). Since hatching eggs were considered as population in the present study, additional data on fertile eggs (number) and hatched chicks (number) were extracted.

*Intervention Details.* Data extracted concerning the intervention included: information on *E. coli* exposure (day of infection, d), any treatment received before infection (day, d and method of application), duration of infection (days), description of the biosecurity measure(s) and the comparator, application of the biosecurity measure(s) and the comparator (method and duration of exposure, in hours), production stage/age of birds when the biosecurity measure(s) and the comparator were applied (day, d). Other extracted information can be found in Supplementary Table 5.

### Outcomes

The outcomes’ data extracted from the eligible studies included: FCR, condemnations at slaughter, and mortality due to colibacillosis. For all these outcomes, data on the number of events (for mortality and condemnations at slaughter), the percentage quantity (for FCR), the production stage/age of birds (days) when the outcome(s) were measured and the duration (start and end, days) of the measured outcome were extracted.

### Effect Measures

During data extraction, both mortality and condemnations at slaughter were recorded as number of mortality/condemnation events among the population considered in the trial. The effect measure used for FCR in data extraction was the mean difference as reported in the original papers.

### Risk of Bias Assessment

Risk of bias was assessed only for controlled trials by using the Cochrane risk-of-bias tool for randomized trials (RoB 2.0) (Higgins et al., 2022) (Supplementary Tables 6–9). The following domains of bias were assessed: bias arising from the randomization process, bias due to deviations from the intended interventions, bias due to missing outcome data, bias in the measurement of the outcome and bias in selection of the reported result.

### Synthesis Methods

Included studies were narratively summarized after extraction and tabulation.

## RESULTS

### Study Selection

**Figure 1:**
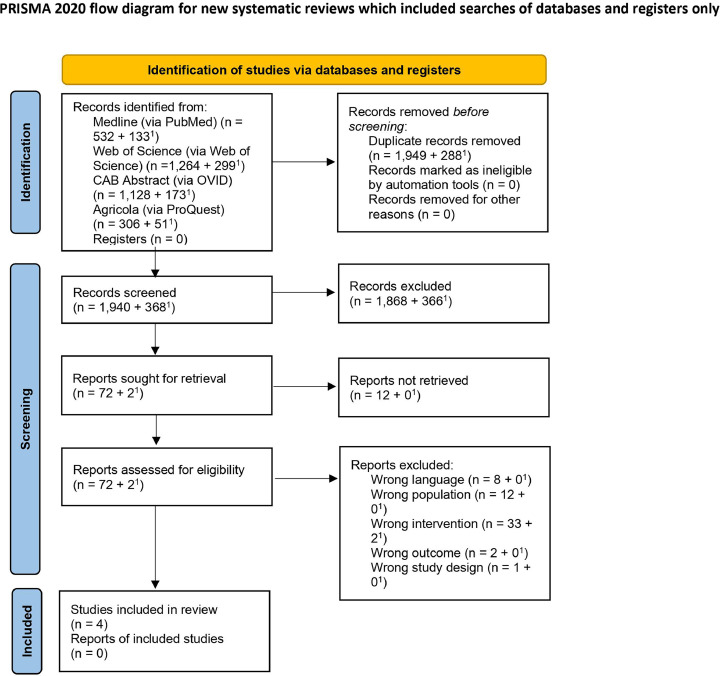
Flow diagram according to PRISMA guidelines showing the selection process for the systematic review on biosecurity to prevent or control colibacillosis in broiler production. ^1^Refers to the number of papers obtained with the additional search run in April 2023.

Out of the 3,886 records identified after the databases search, 2,237 were removed as duplicates and 2,308 were screened for eligibility (Figure 1). During the title and abstract screening, 2,234 records were excluded as not complying with the inclusion criteria (96.8%), while 74 records passed to the full text screening phase. Ultimately, only 4 studies were included as fitting the inclusion criteria (Oosterik et al., 2015; Gholami-Ahangaran et al., 2016; Li et al., 2018; Graham et al., 2021).

After the full-text screening, a considerable number of papers were found to pertain to slaughterhouse practices and food products. Therefore, the authors decided to not consider this level of the production chain, and all the studies performed in the slaughterhouse or considering the food products/carcasses were excluded. Studies found to deal with egg incubators, hatcheries, or eggs were instead included. This distinction was made since the target of the study was to find evidence on the role of biosecurity measures to prevent or control colibacillosis during the rearing cycle. Three additional studies appeared to meet the inclusion criteria but eventually were excluded as not clearly (Toghyani et al., 2020) and/or solely referring to APEC infection, i.e. mix culture of *Staphylococcus aureus* and *E. coli* (Mowry et al., 1980) or multiple pathogens including *E. coli, S. aureus, S. chromogenes, Enterococcus faecalis, Aspergillus fumigatus* (Selby et al., 2023).

### Study Characteristics

Of the studies included, 3 were randomized controlled trials (**RCTs**) in which animals were challenged with *E. coli* (Oosterik et al., 2015; Li et al., 2018; Graham et al., 2021), and one was a RCT with natural disease exposure (Gholami-Ahangaran et al., 2016).

The characteristics of eligible trials are shown in Table 2. All the studies were performed after 2010 and were carried out in Canada (Li et al., 2018), USA (Graham et al., 2021), Belgium (Oosterik et al., 2015), and Iran (Gholami-Ahangaran et al., 2016) in both commercial (Oosterik et al., 2015) and experimental flocks (Li et al., 2018; Graham et al., 2021). One study focused on chicks only (Oosterik et al., 2015) while the other 3 (Gholami-Ahangaran et al., 2016, Li et al., 2018, Graham et al., 2021) focused on eggs (hatching period) and, subsequently, on hatched chicks. Mortality (either embryo or chick) and FCR were the 2 outcomes studied in the selected trials. No information on condemnations at the slaughterhouse was found.

**Table 2:** Characteristics of the eligible studies examining the efficacy of biosecurity to prevent or control colibacillosis in broiler production.

References	Country	Language	Type of study	Strain of birds	Setting	Sex	No. groups	Population studied	Outcome studied
Gholami-Ahangaran et al., 2016	Iran	English	RCT	ND	ND	ND	3	Eggs and chicks	Mortality and FCR
Graham et al*.*, 2021	United States	English	RCT	Ross 308	Experimental flock	ND	4 (two experiments)	Eggs and chicks	Mortality
Li et al., 2018	Canada	English	RCT	Ross 308	Experimental flock	Mixed	3	Eggs and chicks	Mortality and FCR
Oosterik et al., 2015	Belgium	English	RCT	Ross	Commercial flock	ND	2	Chicks	Mortality

All the studies dealt with the same intervention measure, namely disinfection. Since disinfection was assessed either during the incubation/hatching period or directly on the chicks, the original population (broiler) was divided into 2 subgroups consisting of the intervention made in the pre-hatching period (n = 3) or the intervention made in the post-hatching period (n = 1). For the group that received intervention in the pre-hatching period, outcomes were measured in the pre-hatching and in the post-hatching period. Information regarding the intervention during the pre-hatching and the post-hatching period is reported in Table 3.

**Table 3:** Summary of the infection and intervention data concerning the pre- and post-hatching period retrieved from the eligible studies after the screening phase.

References	Population (subset)	Infection with *E. coli* (day)	Treatment applied before infection with *E. coli*	Biosecurity measure	Method of biosecurity measure application	Duration of exposure to the biosecurity measure (minutes)	Age of eggs when intervention was applied (days)
Oosterik et al., 2015	Post-hatching	d31 and d32 with a virulent APEC strain CH2 (10 ml of 10^10^ colony forming units/ml (cfu/ml)) with a compressor and nebulizer	Oral vaccination against Newcastle disease at d28	Exposition of chicks to 10 ml of 1% H_2_O_2_	Nebulization	60	d32-d33
				Exposition of chicks to 10 ml of 2% H_2_O_2_			
Gholami-Ahangaran et al., 2016	Pre-hatching	No artificial contamination of eggs	ND	Exposition of eggs to Virkon S 1/100 dilution	1500 ppm sprayed on each egg. After spraying, fertile eggs remained in room temperature for drying	ND	0, (disinfected in the breeder farm, 2 h after laying)
				Exposition of eggs to Virkon S 1/200 dilution			
				Exposition of eggs to formaldehyde	Disinfected in incubator (under commercial conditions)		
Graham et al*.*, 2021	Pre-hatching	Infected at d19 of incubation with isolate I1 (recovered postmortem from diseased chicks). In ovo administration of either 1.00 × 10^2^ confirmed cfu/200 uL/embryo (Experiment 2) or 1.12 × 10^2^ confirmed cfu/200uL/embryo (Experiment 3)	ND	Exposition of eggs to formaldehyde	Fumigation of the hatch cabinet via a drip application of 6mL of formalin every 3 h after	Every 3 h for ND time, after transfer from the incubator to the hatching cabinet until 12 h before hatch pull	Not clear, (fumigation started after the transfer from the incubator to the hatching cabinet)
Li et al., 2018	Pre-hatching	Infected at d3. post laying . Submerged in a BPW containing 5.7 × 10^6^ cfu / mL NA-resistant *E. coli*	Storage of eggs (after collection on field) at 15°C and 75% relative humidity for two days prior to incubation	Exposition of eggs to 1.5% lysozyme product (Inovapure) (LP)	Fumigated to generate an aerosol (7 to 10 microns) with ultrasonic technology. Order of sanitizer application randomized	10	Three (after collection of eggs on field, storage and before the beginning of incubation)
				Exposition of eggs to 3.0% lysozyme product (Inovapure) (LP)			
				Exposition of eggs to 0.125% quaternary ammonium (QA)			

Except for the study of Graham et al. (2021), where no treatment was applied in the negative control group, the remaining studies used distilled water as a comparator. The duration of the infection was 60 and 1 minute in the studies of Oosterik et al. (2015) and Li et al. (2018), respectively. This information was not provided in any of the other included studies. Oosterik et al. (2015) used APEC CH2 during the experiments, while the serotypes used in the remaining studies were not reported.

Abbreviations: ND, not defined

Concerning the pre-hatching period, the intervention consisted in the exposition of the eggs to different disinfection protocols either after experimental exposure to *E. coli* (Li et al., 2018; Graham et al., 2021) by using nalidixic acid (**NA**)-resistant *E.coli* and wild-type *E.coli*, respectively, or assuming a natural exposure to *E. coli* (Gholami-Ahangaran et al., 2016). The disinfection method was through a spraying system (Gholami-Ahangaran et al., 2016) or by fumigation of the eggs either in the egg incubator (Li et al., 2018) or in the hatcher (Graham et al., 2021). The intervention was applied between the laying and the beginning of the incubation (Gholami-Ahangaran et al., 2016; Li et al., 2018) or between the end of the incubation and the beginning of the hatching phase (Graham et al., 2021).

Concerning the post-hatching period, the intervention dealt with the exposition of chicks to different disinfection protocols (at d 32–33) after experimental exposure to the virulent strain CH2 (at d 31–32) in both cases *via* nebulization directly on chicks (Oosterik et al., 2015). The biosecurity measure was the use of disinfectants effective against viruses, bacteria and fungi (Virkon S and Quaternary ammonium) and yeasts (H_2_O_2_), with antimicrobial characteristics (Formaldehyde) or natural bacteriolytic enzyme (**LP**). In general, the application method included spraying (Gholami-Ahangaran et al., 2016), fumigation (Li et al., 2018; Graham et al., 2021) and nebulization (Oosterik et al., 2015). The duration of the disinfectants’ application varied from 10 (Li et al., 2018) to 60 minutes (Oosterik et al., 2015). Comparisons in 3 studies were performed with groups receiving distilled water (Oosterik et al., 2015; Gholami-Ahangaran et al., 2016; Li et al., 2018). In the remaining study, no intervention was carried out, that is, no fogging of the hatcher (Graham et al., 2021).

### Risk of Bias and Additional Analysis

All the included studies were assessed for bias only for the mortality outcome. Oosterik et al. (2015) presented a very high risk of bias, since there were no measurements of the pre-hatching mortality (or embryo mortality). For all the other 3 studies (Gholami-Ahangaran et al., 2016; Li et al., 2018; Graham et al., 2021), the risk of bias assessment presented some concerns regarding the quality of the evidence. FCR was not assessed as outcome for the risk of bias since it was not measured consistently in most papers. Similarly, condemnations at slaughter were not assessed for bias, since it was not reported as an outcome in any of the included studies.

### Results of Individual Studies

All the studies reported to have either FCR or mortality as outcome. Despite the intervention having been made before hatching in the pre-hatching subgroup, Gholami-Ahangaran et al. (2016), Li et al. (2018), and Graham et al. (2021) assessed the outcomes only in the post-hatching period. Information regarding the outcomes in the pre-hatching period and the post-hatching period are reported in Table 4, Table 5, respectively.

**Table 4:** Summary of the outcome data concerning the pre-hatching period retrieved from the eligible studies after the screening phase.

References	Group ID (number of eggs)	Fertile eggs (number)	Hatched chicks (number)	Biosecurity measure	Period when outcome (mortality) was recorded (days)	Mortality events due to *E. coli* (percentage)
Gholami-Ahangaran et al., 2016	Group 1	30	15	Exposition of eggs to Virkon S 1/100 dilution	From start laying to hatching day (22)	2 (13.33)
	Group 2	30	23	Exposition of eggs to Virkon S 1/200 dilution		1 (4.35)
	Group 3	30	25	Exposition of eggs to formaldehyde gas		0 (0.00)
	Negative Control	30	27	Exposition of eggs to distilled water		3 (11.11)
Li et al., 2018	Group 1 (640 male and 640 female with 4 replicates)	311	289	Exposition of eggs to 1.5% lysozyme product (Inovapure) (LP)	Incubation period (23.3)	21 (6.69)
	Group 2 (640 male and 640 female with 4 replicates)	309	277	Exposition of eggs to 3.0% lysozyme product (Inovapure) (LP)		32 (10.33)
	Group 3 (640 male and 640 female with 4 replicates)	314	282	Exposition of eggs to 0.125% quaternary ammonium (QA)		32 (10.28)
	Negative Control (640 male and 640 female with 4 replicates)	313	281	Exposition of eggs to distilled water		32 (10.26)
Graham et al*.*, 2021	Group 1 (210 eggs) – Experiment 2	ND	Not impacted	Exposition to I1 strain (contact)	Incubation period	Hatchability not impacted (0)
	Group 2 (210 eggs) – Experiment 2			Exposition to I1 strain (seeder)		
	Group 3 (210 eggs) – Experiment 2			Exposition to I1 strain + formaldehyde (contact)		
	Group 4 (210 eggs) – Experiment 2			Exposition to I1 strain + formaldehyde (seeder)		
	Negative control – Experiment 2			ND		
	Group 5 (210 eggs) – Experiment 3			Exposition to I1 strain (contact)		
	Group 6 (210 eggs) – Experiment 3			Exposition to I1 strain (seeder)		
	Group 7 (210 eggs) – Experiment 3			Exposition to I1 strain + formaldehyde (contact)		
	Group 8 (210 eggs) – Experiment 3			Exposition to I1 strain + formaldehyde (seeder)		
	Negative control – Experiment 3			ND		

Only mortality is reported as outcome referring to this subpopulation. Data on FCR and condemnations to the slaughterhouse were not applicable.

Mortality observation period (start day-end day) was as follows: from d 0 of laying to d 22 of hatching (Gholami-Ahangaran et al., 2016), from the start of incubation to d 24.3 of hatching (Li et al., 2018), and from the starting of incubation to hatching day (Graham et al*.*, 2021).

Abbreviation: ND, not defined

**Table 5:** Summary of the outcome data concerning the post-hatching period retrieved from the eligible studies after the screening phase. Mortality and FCR are reported as outcomes referring to this subpopulation.

References	Group ID (number of animals/eggs)	Biosecurity measure	Period when outcomes (mortality and FCR) were recorded	Mortality or number of dead (percentage)	FCR (feed/gain)
Oosterik et al., 2015	Group 1 (5 chicks)	Exposition of chicks to 10 mL of 1% H_2_O_2_	From 1 to 7-8 d	0	NA
	Group 2 (5 chicks)	Exposition of chicks to 10 mL of 2% H2O2		0	
	Negative control (5 chicks)	Exposition of chicks to distilled water		0	
Gholami-Ahangaran et al., 2016	Group 1 (30 eggs with 3 replicates-15 chicks hatched)	Exposition of eggs to Virkon S 1/100 dilution	Hatching day until 7 d post hatching	1 (6.67)	0.81
	Group 2 (30 eggs with 3 replicates-23 chicks hatched)	Exposition of eggs to Virkon S 1/200 dilution		1 (4.35)	0.80
	Group 3 (30 eggs with 3 replicates-25 chicks hatched)	Exposition of eggs to formaldehyde gas		1 (4)	0.82
	Negative Control (30 eggs with 3 replicates-27 chicks hatched)	Exposition of eggs to distilled water		13 (48.15)	0.81
Li et al., 2018	Group 1 (640 male and 640 female with 4 replicates)	Exposition of eggs to 1.5% lysozyme product (Inovapure) (LP)	From d 1 to 33 d	3 (2.2)	1.43
	Group 2 (640 male and 640 female with 4 replicates)	Exposition of eggs to 3.0% lysozyme product (Inovapure) (LP)		3 (2.2)	1.43
	Group 3 (640 male and 640 female with 4 replicates)	Exposition of eggs to 0.125% quaternary ammonium (QA)		4 (2.5)	1.40
	Negative Control (640 male and 640 female with 4 replicates)	Exposition of eggs to distilled water		5 (3.1)	1.42
Graham et al*.*, 2021	Group 1 (210 eggs) – Experiment 2	Exposition to I1 strain (contact)	From day of hatch to 7 d	Not impacted	NA
	Group 2 (210 eggs) – Experiment 2	Exposition to I1 strain (seeder)		Not impacted	NA
	Group 3 (210 eggs) – Experiment 2	Exposition to I1 strain + formaldehyde (contact)		Not impacted	NA
	Group 4 (210 eggs) – Experiment 2	Exposition to I1 strain + formaldehyde (seeder)		Not impacted	NA
	Negative control – Experiment 2	ND		Not impacted	NA
	Group 5 (210 eggs) – Experiment 3	Exposition to I1 strain (contact)		Not impacted	NA
	Group 6 (210 eggs) – Experiment 3	Exposition to I1 strain (seeder)		Not impacted	NA
	Group 7 (210 eggs) – Experiment 3	Exposition to I1 strain + formaldehyde (contact)		Not impacted	NA
	Group 8 (210 eggs) – Experiment 3	Exposition to I1 strain + formaldehyde (seeder)		Not impacted	NA
	Negative control – Experiment 3	ND		Not impacted	NA

Abbreviations: ND, not defined; NA, not applicable.

Concerning the pre-hatching period (Table 4), embryo mortality was the only outcome retrievable (Gholami-Ahangaran et al., 2016; Li et al., 2018; Graham et al., 2021). In detail, the embryo mortality was recorded during the whole incubation period (from freshly laid eggs to hatching), accounting for 22 d (Gholami-Ahangaran et al., 2016) or 23.3 d (Li et al., 2018). Embryo mortality was calculated upon the number of hatched chicks in both studies. Formaldehyde disinfection by spraying (Gholami-Ahangaran et al., 2016) and disinfection with 1.5% lysozyme product (**LP**) by fumigation (Li et al., 2018) obtained the lowest embryo mortality among the tested protocols, while according to Graham et al. (2021), the hatchability was not impacted as result of the challenge.

Referring to the post-hatching period (Table 5), chick mortality was recorded in all the studies, while FCR was recorded only in 2 studies (Gholami-Ahangaran et al., 2016; Li et al., 2018). Specifically, the observation period for all the outcomes was until d 7 to 8 d post-hatch (Gholami-Ahangaran et al., 2016; Li et al., 2018; Graham et al., 2021) or until the end of the rearing cycle (Oosterik et al., 2015) accounting for 7 to 8 d and 33 d, respectively. In the observation period, chick mortality was neither impacted according to Graham et al. (2021) nor observed according to Oosterik et al. (2015), but it was higher in the control group than the treated groups in Gholami-Ahangaran et al. (2016) and Li et al. (2018). In the observation period, FCR was slightly higher in the formaldehyde treated group (Gholami-Ahangaran et al., 2016) and in the 2 lysozyme product (LP) groups (Li et al., 2018) than in the other tested groups.

### Results of the Synthesis

The aim of this systematic review was to conduct a quantitative synthesis of results *via* a (network) meta-analysis. The synthesis approach proposed in the protocol was not conducted because of the few eligible trials and the scarcity of data available to address the research question. Additionally, a meta-analysis and sensibility analysis were not performed. However, trial results are presented in Tables 2 to 5. No summary measure was calculated and heterogeneity was not formally assessed.

### Reporting Biases

All the studies were assessed for biases concerning only mortality as outcome. All the studies were judged to have some bias concerns for multiple domains (i.e., bias arising from the randomization process, bias due to deviations from the intended interventions, bias in the measurement of the outcome and bias in selection of the reported result) in a way that substantially lowered the confidence of results. Gholami-Ahangaran et al. (2016), Li et al. (2018), and Graham et al. (2021) reported to have a low risk concerning bias due to missing outcome data, while Oosterik et al. (2015) was judged to be at high risk due to missing outcome as there was no pre-hatching phase. Additionally, lack of details concerning the relationship between the exposure to the pathogen and the application of the intervention was found in all the studies. Results of the risk of bias assesment are presented as Supplementary Material 6-9.

### Certainty of Evidence

No assessment of certainty was performed due to the limited number of papers included.

## DISCUSSION

Biosecurity measures to prevent infectious diseases are crucial for controlling animal health, antimicrobial use, and public health issues in livestock production, including poultry (Hulme, 2021; Mallioris et al., 2023). Despite its importance, the role of biosecurity in colibacillosis prevention or control has never been thoroughly investigated even considering the high impact of the disease on the poultry industry. Thus, the purpose of this systematic review was to investigate the role that biosecurity measures may have in preventing or controlling colibacillosis in broiler production. The findings included only 4 studies that matched the criteria set in the original protocol (Oosterik et al., 2015; Gholami-Ahangaran et al., 2016; Li et al., 2018; Graham et al., 2021).

The term “biosecurity” used in the search served as an umbrella term, encompassing all measures related to preventing the entry and spread of pathogens to and within the farm (WOAH, 2022). Consequently, all the measures referred to the aforementioned practices should be considered, ranging from the entry of visitors, vehicles, and animals in the farm, location of the farm (external biosecurity) to cleaning and disinfection procedures, measures between different farm compartments and eggs management (internal biosecurity) (Gelaude et al., 2014; Van Limbergen et al., 2018; Delpont et al., 2021). Given the diversity and the large number of practices related to biosecurity and the difficulty, in some cases, to distinguish between biosecurity and management interventions, an overlap between the two topics were to be expected (Kumar and Bhattacharya, 2019). For this systematic review, a panel of poultry experts from the poultry colibacillosis drafting group of ENOVAT was consulted to provide their opinion on the characteristics of the PICO elements and the search string. The feedback received was subsequently discussed among the authors, who collectively decided on the final search string. Thus, the search terms related to [Biosecurity] were validated after expert consultation, to define which practices were specifically related to it. As a consequence, the adopted list of keywords related to biosecurity aimed at being as comprehensive as possible, allowing us to capture the complexity and broadness of the topic in the reviewed studies.

Despite the heterogeneity of terms related to biosecurity that could have potentially led to different studies, only RCTs investigating exclusively the role of disinfection either in the pre-hatching or in the post-hatching phase for colibacillosis prevention or control, were retrieved (Table 2). Disinfection in the pre-hatching period is a fundamental practice to prevent bacterial outbreaks in the hatchery (Motola et al., 2023), but it is only one out of the several biosecurity measures that can be implemented.

Regarding the findings obtained in this systematic review, Gholami-Ahangaran et al. (2016) compared the use of disinfection with formaldehyde with other two alternative protocols, resulting in better outcomes in terms of mortality (both embryos and chicks) but in slightly worst effect on FCR (Table 4, Table 5). Similarly, Graham et al. (2021) reported no impact on hatchability and mortality after fumigation of the hatching room (Table 4, Table 5). This finding is in line with available literature (Motola et al., 2023). Among the used disinfectants and disinfection techniques, fumigation with formaldehyde is the gold standard in the hatchery environment. However, it can result in toxic residues and compromise the hatchability and the chicks growth performance (Oliveira et al., 2020); hence it represents a high risk for chick quality and personnel's health (Çadırcı, 2009; Duong et al., 2011). Recent studies assessed different alternatives, both natural (e.g., use of probiotics, eucalyptus extract, essential oils) (Graham et al., 2018; Motola et al., 2020; Toghyiani et al., 2020) and chemical (e.g., hydrogen peroxide, peracetic acid, low energy electron beam) (Motola et al., 2020; Pees et al., 2022), depicting a general interest in finding alternative disinfection protocols for hatching eggs and suggesting awareness and/or commercial interest on alternative solutions.

Among the included papers, Li et al. (2018) tested different disinfection protocols with different disinfectants, namely lysozyme product (**LP**) at different concentrations and quaternary ammonium (**QA**) (Table 3). This intervention resulted in better effect on mortality due to colibacillosis by fumigating the eggs in the pre-hatching phase with 1.5% LP and better results in FCR by fumigating the eggs in the pre-hatching phase with 0.125% QA (Table 4, Table 5).

Another possible reason explaining the low number of papers included could be related to the typology of studies, that is, only RCTs eligible to assess the role of biosecurity to prevent or control colibacillosis. All the included studies compared the effects on a randomly allocated population of different disinfection protocols. RCTs with specific use of disinfectants are linked to procedural biosecurity (e.g., disinfection protocols) and are easier to test than trials linked to structural biosecurity (e.g., the role of having *vs.* not having the farm fencing, for example), which may need a different study design to be investigated. Hence, addressing experimental design challenges concerning relevant structural biosecurity measures can be complex due to various factors (e.g., APEC serotype, animal breed), which could potentially influence the final results. Similar challenges have been documented in other reviews (Paudel et al., 2024). However, the risk of bias assessment judged all the aforementioned studies as presenting some concerns related to the randomization process, suggesting potential challenges encountered in the RCT design for the evidence-based veterinary medicine (Vandeweerd et al. 2012).

The results of the included studies in this systematic review were difficult to compare due to the heterogeneity of information described (i.e., different protocols, disinfectants, biosecurity measures, outcomes, population subsets). For example, contrary to the other included studies, Oosterik et al. (2015) assessed the use of disinfectants directly on chicks (post-hatching population) to lower APEC pathogenicity (so potentially to control colibacillosis), highlighting that the use of H_2_O_2_ as disinfectant nebulized directly on chicks is not advisable since the susceptibility to APEC infection increases rather than decreases.

According to the risk of bias assessment, trustworthiness of the included studies is limited because they presented some concerns regarding multiple bias domains. Specifically, Oosterik et al. (2015) was judged as carrying high risk of bias related to missing outcome data. However, the risk of bias tool used mostly assessed parameters that are of interest in RCTs involving human trials. Therefore, this assessment seems to be not particularly informative in studies as those described in this paper, highlighting the need for dedicated risk of bias tools in veterinary medicine reviews.

In this review, the outcomes were selected according to stakeholders’ consultation (similarly to the selection of the keyword terms), and only mortality, FCR, and condemnations at slaughter due to colibacillosis were considered. However, during the screening process of the eligible studies, other outcomes such as lesion scoring, body weight gain, hatchability of eggs were repetitively reported, with lesion scoring being the most frequent one, as also reported in Oosterik et al. (2015). This may be a suggestion for improving the search methodology in the future since the aforementioned outcomes can be of interest for stakeholders as well. The notable lack of direct study evidence linking specific biosecurity practices to outcomes associated with APEC and infected flock might be attributed to the challenges in designing studies to assess biosecurity measures. Additionally, as reported by Delpont et al. (2023), the absence of available data on biosecurity may have contributed to the difficulties encountered in retrieving suitable studies for this systematic review.

The scarcity of studies matching the selection criteria, in addition to the broadness of the topic, highlights the urgent need for further research. Specifically, studies investigating the relationship between the implementation of biosecurity measures and the incidence of colibacillosis would be highly valuable. Such studies are necessary to accurately assess the impact of biosecurity in preventing the disease.

## CONCLUSIONS

This systematic review aimed at addressing the role of biosecurity to prevent or control colibacillosis in the broiler production chain by screening all available and eligible studies and therefore contributing to the evidence-based decision-making process when adopting specific biosecurity measures. The scarcity of available eligible studies, together with the broadness of the biosecurity concept, reflected by the diversity of interventions applied, highlight the absence of supportive evidence for the efficacy of biosecurity measures to prevent or control colibacillosis in broilers. Therefore, additional research addressing this gap is strongly needed.

## DISCLOSURES

The authors declare no conflicts of interest.

## Appendix Supplementary materials

Image, application 1

Image, application 2

Image, application 3

Image, application 4

Image, application 5

Image, application 6

Image, application 7

Image, application 8

Image, application 9

## Footnotes

Supplementary material associated with this article can be found in the online version at doi:10.1016/j.psj.2024.103955.

## References

[bib0001] Ahamed Y.I., Adikari A.M., Gamlath G.A., Somarathna W.A. (2019). Effects of floor and nest eggs on hatchability and chick quality parameters in broiler breeders. WJAS.

[bib0002] Apostolakos I., Laconi A., Mughini-Gras L., Yapicier Ö.Ş., Piccirillo A. (2021). Occurrence of Colibacillosis in Broilers and Its Relationship With Avian pathogenic Escherichia coli (APEC) population structure and molecular characteristics. Front. Vet. Sci..

[bib0003] Bramer W.M., Rethlefsen M.L., Kleijnen J., Franco O.H. (2017). Optimal database combinations for literature searches in systematic reviews: a prospective exploratory study. Syst. Rev..

[bib0004] Çadırcı S. (2009). Disinfection of hatching eggs by formaldehyde fumigation a review. Arch.Geflügelk.

[bib0005] Christensen H., Bachmeier J., Bisgaard M. (2021). New strategies to prevent and control avian pathogenic Escherichia coli (APEC). Avian Pathol.

[bib0006] Delpont M., Guinat C., Guérin J.L., Le leu E., Vaillancourt J.P., Paul M.C. (2021). Biosecurity measures in French poultry farms are associated with farm type and location. Prev. Vet. Med..

[bib0007] Delpont M., Salazar L.G., Dewulf J., Zbikowski A., Szeleszczuk P., Lefort A.C., Rousset N., Spaans A., Amalraj A., Tilli G., Piccirillo A., Devesa A., Sevilla-Navarro S., van Meirhaege H., Kovacs L., Jozwiak A.B., Guérin J.L., Paul M.C. (2023). Monitoring biosecurity in poultry production: an overview of databases reporting biosecurity compliance from seven European countries. Prev. Vet. Med..

[bib0008] Duong A., Steinmaus C., McHale C.M., Vaughan C.P., Zhang L. (2011). Reproductive and developmental toxicity of formaldehyde: a systematic review. Mutat. Res-Rev. Mutat..

[bib0009] Eijck, I., and J. De Wilt. 2009. Co-innovatieprogramma Landbouwhuisdieren en Antibiotica Reductie (CLEAR). Accessed Apr. 2023. https://edepot.wur.nl/12903

[bib0010] Gelaude P., Schlepers M., Verlinden M., Laanen M., Dewulf J. (2014). Biocheck.UGent: a quantitative tool to measure biosecurity at broiler farms and the relationship with technical performances and antimicrobial use. Poult. Sci..

[bib0011] Gholami-Ahangaran M., Shahzamani S., Yazdkhasti M. (2016). Comparison of Virkon S (R) and Formaldehyde on hatchability and survival rate of chicks in disinfection of fertile eggs. Revue. Médecine Vétérinaire..

[bib0012] Giovanardi D., Campagnari E., Sperati Ruffoni L., Pesente P., Ortali G., Furlattini V. (2005). Avian pathogenic Escherichia coli transmission from broiler breeders to their progeny in an integrated poultry production chain. Avian Pathol.

[bib0013] Graham B.D., Selby C.M., Graham L.E., Teague K.D., Tellez-Isaias G., Hargis B.M., Vuong C.N. (2021). Development of a wild-type Escherichia coli environmental bloom model to evaluate alternatives to formaldehyde fumigation in broiler chicken hatch cabinets. Poult. Sci..

[bib0014] Graham L.E., Teague K.D., Latorre J.D., Yang Y., Baxter M.F.A., Mahaffey B.D., Hernandez-Velasco X., Bielke L.R., Hargis B.M., Tellez G. (2018). Use of probiotics as an alternative to formaldehyde fumigation in commercial broiler chicken hatch cabinets. J. Appl. Poultry Res..

[bib0015] Guabiraba R., Schouler C. (2015). Avian colibacillosis: still many black holes. FEMS Microbiol. Lett..

[bib0016] Helmy Y.A., Deblais L., Kassem I.I., Kathayat D., Rajashekara G. (2018). Novel small molecule modulators of quorum sensing in avian pathogenic Escherichia coli (APEC). Virulence.

[bib0017] Higgins, J. P. T., J. Thomas, J. Chandler, M. Cumpston, T. Li, M. J. Page, and V. A. Welch. 2022. Cochrane handbook for systematic reviews of interventions version 6.3. Accessed Apr. 2023. https://training.cochrane.org/handbook/current

[bib0018] Himsworth C.G., Zabek E., Desruisseau A., Parmley E.J., Reid-Smith R., Leslie M., Ambrose N., Patrick D.M., Cox W. (2016). Avian pathogenicity genes and antibiotic resistance in Escherichia coli isolates from wild Norway rats (Rattus norvegicus) in British Columbia. Canada. J. Wildlife Dis..

[bib0019] Hulme P.E. (2021). One biosecurity: a unified concept to integrate human, animal, plant, and environmental health. Emerg. Top. Life Sci..

[bib0020] Kathayat D., Lokesh D., Ranjit S., Rajashekara G. (2021). Avian pathogenic Escherichia Coli (Apec): an overview of virulence and pathogenesis factors, zoonotic potential, and control strategies. Pathogens.

[bib0021] Kumar I., Bhattacharya J. (2019). Assessment of the role of silver nanoparticles in reducing poultry mortality, risk and economic benefits. Appl. Nanosci..

[bib0022] Landman W.J., Van Eck J.H. (2015). The incidence and economic impact of the Escherichia coli peritonitis syndrome in Dutch poultry farming. Avian Pathol.

[bib0023] Li X., Anderson D., Rathgeber B., McLean N., MacIsaac J. (2018). Fumigating broiler hatching eggs with lysozyme product (Inovapure) to reduce eggshell microbial load. Poult. Sci..

[bib0024] Lutful Kabir S.M. (2010). Avian colibacillosis and salmonellosis: a closer look at epidemiology, pathogenesis, diagnosis, control and public health concerns. Int. J. Environ. Res. Public Health..

[bib0025] Mallioris P., Teunis G., Lagerweij G., Joosten P., Dewulf J., Wagenaar J.A., Stegeman A., Mughini-Gras L. (2023). Biosecurity and antimicrobial use in broiler farms across nine European countries: toward identifying farm-specific options for reducing antimicrobial usage. Epidemiol. Infect..

[bib0026] Mehat J.W., Arnoud van Vliet H.M., La Ragione R.M. (2021). The Avian Pathogenic Escherichia coli (APEC) pathotype is comprised of multiple distinct, independent genotypes. Avian Pathol.

[bib0027] Motola G., Hafez H.M., Brüggemann-Schwarze S. (2020). Efficacy of six disinfection methods against extended-spectrum beta-lactamase (ESBL) producing E. coli on eggshells in vitro. PloS One.

[bib0028] Motola G., Hafez H.M., Brüggemann-Schwarze S. (2023). Assessment of three alternative methods for bacterial disinfection of hatching eggs in comparison with conventional approach in commercial broiler hatcheries. PloS One.

[bib0029] Mowry D.J., Fagerberg D.J., Quarles C.L. (1980). Effect of hatcher fogging on hatcher airborne bacteria and broiler performance. Poult. Sci..

[bib0030] Newman D.M., Barbieri N.L., de Oliveira A.L., Willis D., Nolan L.K., Logue C.M. (2021). Characterizing avian pathogenic Escherichia coli (APEC) from colibacillosis cases, 2018. PeerJ.

[bib0031] Nguyen T.T.T., Allan B., Wheler C., Köster W., Gerdts V., Dar A. (2021). Avian antimicrobial peptides: in vitro and in ovo characterization and protection from early chick mortality caused by yolk sac infection. Sci. Rep..

[bib0032] Nolan L.K., Vaillancourt J.P., Barbieri N., Logue C.M., Swayne D.E., Boulianne M., Logue C.M., McDougald L.R., Nair V.L., Suarez D.L. (2020). Pages 770–830 in Diseases of Poultry.

[bib0033] Oliveira G.D.S., Dos Santos V.M., Nascimento S.T., Rodrigues J.C. (2020). Alternative sanitizers to paraformaldehyde for incubation of fertile eggs. Poult. Sci..

[bib0034] Oosterik L.H., Tuntufye H.N., Janssens S., Butaye P., Goddeeris B.M. (2015). Disinfection by hydrogen peroxide nebulization increases susceptibility to avian pathogenic Escherichia coli. BMC Res. Notes..

[bib0035] Ozaki H., Matsuoka Y., Nakagawa E., Murase T. (2017). Characteristics of Escherichia coli isolated from broiler chickens with colibacillosis in commercial farms from a common hatchery. Poult. Sci..

[bib0036] Page M.J., Mckenzie J.E., Bossuyt P.M., Boutron I., Hoffmann T.C., Mulrow C.D., Shamseer L., Tetzlaff J.M., Akl E.A., Brennan S.E., Chou R., Glanville J., Grimshaw J.M., Hróbjartsson A., Lalu M.M., Li T., Loder E.W., Mayo-Wilson E., Mcdonald S., McGuinness L.A., Stewart L.A., Thomas J., Tricco A.C., Welch V.A., Whiting P., Moher D. (2021). The PRISMA 2020 statement: an updated guideline for reporting systematic reviews. BMJ.

[bib0037] Paudel S., Apostolakos I., Vougat Ngom R., Tilli G., de Carvalho Ferreira H., Piccirillo A. (2024). A systematic review and meta-analysis on the efficacy of vaccination against colibacillosis in broiler production. PLoS One.

[bib0038] Pees M., Motola G., Brüggemann-Schwarze S., Bachmeier J., Hafez H.M., Tebrün W. (2022). Impact on hatchability and broiler performance after use of hydrogen peroxide nebulization versus formaldehyde fumigation as pre-incubation hatching egg disinfectants in field trial. Poultry.

[bib0039] Poulsen L.L., Thøfner I., Bisgaard M., Christensen J.P., Olsen R.H., Christensen H. (2017). Longitudinal study of transmission of Escherichia coli from broiler breeders to broilers. Vet. Microbiol..

[bib0040] Sanguinetti, V. M., H. Ganshorn, S. Agbese, and M. C. Windeyer. 2021. Protocol for a systematic review of disease control strategies used to prevent infectious mortality and morbidity in pre-weaned beef calves. PRISM Repository. Accessed Apr. 2023. https://prism.ucalgary.ca/items/73051c3c-6302-4f6e-a349-973f80243a9e

[bib0041] Sarfraz M., Nguyen T.T.T., Wheler C., Köster W., Gerdts V.D.A. (2022). Characterization of dosage levels for in ovo administration of innate immune stimulants for prevention of yolk sac infection in chicks. Vet. Sci..

[bib0042] Schiavone A., Pugliese N., Circella E., Camarda A. (2020). Association between the poultry red mite Dermanyssus gallinae and potential avian pathogenic Escherichia coli (APEC). Vet. Parasitol..

[bib0043] Selby C.M., Beer L.C., Forga A.J., Coles M.E., Graham L.E., Teague K.D., Tellez-Isaias G., Hargis B.M., Vuong C.N., Graham B.D. (2023). Evaluation of the impact of formaldehyde fumigation during the hatching phase on contamination in the hatch cabinet and early performance in broiler chickens. Poult. Sci..

[bib0044] Tamburro M., Sammarco M.L., Trematerra P., Colacci M., Ripabelli G. (2022). Alphitobius diaperinus Panzer (Insecta, Coleoptera) in a single house of a broiler production facility as a potential source of pathogenic bacteria for broilers and humans. Lett. Appl. Microbiol..

[bib0045] Toghyani P., Shahzamani S., Gholami-Ahangaran M., Firouzabadi S.A.M. (2020). Comparison of eucalyptus extract and formaldehyde on hatchability and survival rate of chicks in disinfection of fertile eggs. IJPRAS.

[bib0046] Van Limbergen T., Dewulf J., Klinkenberg M., Ducatelle R., Gelaude P., Méndez J., Heinola K., Papasolomontos S., Szeleszczuk P., Maes D. (2018). Scoring biosecurity in European conventional broiler production. Poult. Sci..

[bib0047] Vandeweerd J.-M., Kirschvink N., Clegg P., Vandenput S., Gustin P., Saegerman C. (2012). Is evidence-based medicine so evident in veterinary research and practice? History, obstacles and perspectives. Vet. J..

[bib0048] World Organisation for Animal Health (WOAH). 2022. Terrestrial animal health code, Glossary. Accessed May 2023. https://www.woah.org/en/what-we-do/standards/codes-and-manuals/terrestrial-code-online-access/?id=169&L=1&htmfile=glossaire.htm

[bib0049] Zhao S., Wang C.-L., Chang S.-K., Tsai Y.-L., Chou C.-H. (2018). Characterization of Escherichia coli isolated from day-old chicken fluff in taiwanese hatcheries. Avian Dis.

