# Supplementary material for: A systematic review on the role of biosecurity to prevent or control colibacillosis in broiler production
Source: Poult Sci. 2024 Jun 6;103(8):103955. doi: 10.1016/j.psj.2024.103955 (PMC11255943; doi:10.1016/j.psj.2024.103955)
Supplement: Supplementary file 3 [file mmc3.docx]

Supplementary Table 3: search strategy applied in Agricola (via ProQuest) for the original and additional search run in November 2021 and April 2023, respectively.

TS=("chicken*" OR "poultry*" OR "flock*" OR "gallus" OR "broiler*") AND TS=("Biosecurity" OR "Clean*" OR "Disinfect*" OR "Disinfest*" OR "Pest " OR "Insect*" OR "Vermin*" OR "Rodent*" OR "Fomites " OR "Sanit*" OR "Hygien*" OR "All in-all out" OR "Downtime" OR "Turnaround" OR "Biological break" OR "Filter zone " OR "Danish entry system" OR "Footdips" OR "Visitor*" OR "Thinning" OR "Depopulation") AND TS=("colibacillosis" OR "colisepticaemia" OR "peritonitis" OR "coli" OR "Escherichia" OR "coliform" OR "colisepticemia" OR "coligranuloma" OR "Hjarre’s" OR "air sac disease" OR "cellulitis" OR "osteomyelitis" OR "brittle bone disease" OR "salpingitis" OR "synovitis" OR "omphalitis" OR "enteritis" OR "hemorrhagic septicemia" OR "chronic respiratory disease" OR "swollen head syndrome" OR "venereal colibacillosis" OR "coliform cellulitis" OR "yolk sac infection" OR "APEC" OR "pathogenic E. coli" OR "primary infection" OR "secondary infection" OR "multifactorial" OR "multicausal")
