# Supplementary material for: A systematic review on the role of biosecurity to prevent or control colibacillosis in broiler production
Source: Poult Sci. 2024 Jun 6;103(8):103955. doi: 10.1016/j.psj.2024.103955 (PMC11255943; doi:10.1016/j.psj.2024.103955)
Supplement: Supplementary file 4 [file mmc4.docx]

Supplementary Table 4: search strategy applied in CAB abstract (via Ovid)) for the original and additional search run in November 2021 and April 2023, respectively.

First search - Nov 1^st^, 2021

CAB Abstracts <1973 to 2021 Week 43>

1 ("chicken*" or "poultry*" or "flock*" or "gallus" or "broiler*").ti,ab. 231354

2 ("Biosecurity" or "Clean*" or "Disinfect*" or "Disinfest*" or "Pest " or "Insect*" or "Vermin*" or "Rodent*" or "Fomites " or "Sanit*" or "Hygien*" or "All in-all out" or "Downtime" or "Turnaround" or "Biological break" or "Filter zone " or "Danish entry system" or "Footdips" or "Visitor*" or "Thinning" or "Depopulation").ti,ab. 645455

3 ("colibacillosis" or "colisepticaemia" or "peritonitis" or "coli" or "Escherichia" or "coliform" or "colisepticemia" or "coligranuloma" or "Hjarre’s" or "air sac disease" or "cellulitis" or "osteomyelitis" or "brittle bone disease" or "salpingitis" or "synovitis" or "omphalitis" or "enteritis" or "hemorrhagic septicemia" or "chronic respiratory disease" or "swollen head syndrome" or "venereal colibacillosis" or "coliform cellulitis" or "yolk sac infection" or "APEC" or "pathogenic E. coli" or "primary infection" or "secondary infection" or "multifactorial" or "multicausal").ti,ab. 173733

4 1 and 2 and 3 1128

Second search – Apr 13^th^, 2023

CAB Abstracts <1973 to 2023 Week 15>

1 ("chicken*" or "poultry*" or "flock*" or "gallus" or "broiler*").ti,ab. 248525

2 ("Biosecurity" or "Clean*" or "Disinfect*" or "Disinfest*" or "Pest " or "Insect*" or "Vermin*" or "Rodent*" or "Fomites " or "Sanit*" or "Hygien*" or "All in-all out" or "Downtime" or "Turnaround" or "Biological break" or "Filter zone " or "Danish entry system" or "Footdips" or "Visitor*" or "Thinning" or "Depopulation").ti,ab. 697727

3 ("colibacillosis" or "colisepticaemia" or "peritonitis" or "coli" or "Escherichia" or "coliform" or "colisepticemia" or "coligranuloma" or "Hjarre’s" or "air sac disease" or "cellulitis" or "osteomyelitis" or "brittle bone disease" or "salpingitis" or "synovitis" or "omphalitis" or "enteritis" or "hemorrhagic septicemia" or "chronic respiratory disease" or "swollen head syndrome" or "venereal colibacillosis" or "coliform cellulitis" or "yolk sac infection" or "APEC" or "pathogenic E. coli" or "primary infection" or "secondary infection" or "multifactorial" or "multicausal").ti,ab. 190383

4 1 and 2 and 3 1272

5 limit 4 to yr="2021 -Current" 173
